# Mind-Body Treatment for International English-Speaking Adults With Neurofibromatosis via Live Videoconferencing: Protocol for a Single-Blind Randomized Controlled Trial

**DOI:** 10.2196/11008

**Published:** 2018-10-23

**Authors:** Ana-Maria Vranceanu, Emily L Zale, Christopher J Funes, Eric A Macklin, Jessica McCurley, Elyse R Park, Justin T Jordan, Ann Lin, Scott R Plotkin

**Affiliations:** 1 Integrated Brain Health Clinical and Research Program Department of Psychiatry Massachusetts General Hospital Boston, MA United States; 2 Biostatistics Center Department of Medicine Massachusetts General Hospital Boston, MA United States; 3 Benson Henry Institute for Mind Body Medicine Department of Psychiatry Massachusetts General Hospital Boston, MA United States; 4 Neurofibromatosis Clinic Department of Neurology Massachusetts General Hospital Boston, MA United States

**Keywords:** neurofibromatosis, quality of life, stress management, mind-body, videoconferencing, resiliency, mobile phone

## Abstract

**Background:**

Neurofibromatoses (NF) are rare genetic conditions associated with substantial psychosocial burden and impaired quality of life (QoL). We developed the first NF-tailored mind-body program (the Relaxation Response Resiliency Program for NF) and adapted it for delivery via live videoconferencing to decrease participation barriers and increase its reach. In a pilot randomized controlled trial (RCT), we found that the Relaxation Response Resiliency Program for NF had excellent feasibility and acceptability when delivered via live videoconferencing; furthermore, the Relaxation Response Resiliency Program for NF showed proof of concept in improving QoL compared with an NF-tailored health education control program (the Health Enhancement Program for NF). A fully powered trial is needed to ascertain the efficacy and durability of the Relaxation Response Resiliency Program for NF delivered via secure live videoconferencing among geographically diverse patients.

**Objective:**

The objective of this study is to evaluate the efficacy of the Relaxation Response Resiliency Program for NF versus the Health Enhancement Program for NF, both delivered in groups via secure live videoconferencing, among geographically diverse patients with NF across the United States and internationally. Here we describe the protocol, manualized treatments, evaluation plan, and study design.

**Methods:**

This is a single-blind RCT. Patients are told that they will be randomized to one of the two stress management programs (stress management program 1: the Relaxation Response Resiliency Program for NF and stress management program 2: the Health Enhancement Program for NF). Patients are recruited from NF-specific national and international foundations and NF clinics across the United States through study ads and a video of participants who have completed the program as part of the pilot study or ongoing trial. Interested participants are screened for eligibility via secure live videoconferencing (self-reported stress and difficulties coping, no change in antidepressant medication within the past 3 months, no psychotherapy within the past 3 months, no major upcoming surgeries within the next 12 months, English speaking, and able to complete questionnaires online and participate in live video interventions) and consent obtained before participation. Both programs are manualized comprising 8 sessions delivered via secure live videoconferencing by trained clinical psychologists. Primary outcomes are physical health QoL and psychological health QoL. Secondary outcomes are social relationship QoL, environment QoL, and psychosocial and resiliency variables. Outcomes are assessed at baseline, posttraining, and 6- and 12-month follow-ups.

**Results:**

The trial is ongoing. Thus far, we have recruited 55 patients and aim to recruit a total of 224. Recruitment will close in May 2020; we plan to complete data analyses by June 2021.

**Conclusions:**

This trial will answer key questions about the efficacy and durability of the Relaxation Response Resiliency Program for NF via live videoconferencing with English-speaking adults with NF worldwide. If found efficacious, this program can be readily implemented through national and international NF foundations and NF-specific clinics. The virtual model of delivery has extensive applications for patients in rural areas, those with disability or illness that precludes travel to clinics, and those with rare diseases.

**Trial Registration:**

ClinicalTrials.gov NCT03406208; https://clinicaltrials.gov/ct2/show/NCT03406208 (Archived by WebCite at http://www.webcitation.org/72ZoTDQ6h)

**International Registered Report Identifier (IRRID):**

RR1-10.2196/11008

## Introduction

Neurofibromatoses (NF) are the most common genetic neurological conditions worldwide and affect men and women of all races and ethnic groups [1,2]. NF comprises 3 genetically distinct conditions (NF1, NF2, and Schwannomatosis) unified by the predisposition to nerve sheath tumors that tend to be histologically benign. Each NF type has characteristic symptoms: NF1 is typically associated with disfiguring cutaneous tumors [3,4]; NF2 is associated with hearing loss, facial weakness, and poor gait [5]; and Schwannomatosis is associated with chronic disabling pain [6]. There is no cure for NF; treatment is limited to symptom management through surgical and palliative means [7].

Despite their distinct pathophysiology, patients’ psychosocial profile is similar regardless of the NF type [8]. As a group, patients with NF have lower quality of life (QoL) and experience more pain compared with general population norms [8,9]. Moreover, rates of depression, anxiety, and stress among patients with NF are comparable to those among patients with cancer and coronary heart disease [9,10]. Despite this heavy psychological burden, there are no evidence-based psychosocial treatments that directly address the specific needs of this population.

We developed the first psychosocial treatment designed to meet the specific needs of patients with NF using a group format. Using a sequential approach that included focus group discussions; in-person open-pilot testing with exit interviews (ie, semistructured interviews conducted by the study therapist after the participants completed the program); and a preliminary randomized controlled trial (RCT) [11], we adapted an evidence-based mind-body intervention [12] to the specific needs of patients with NF and for live video delivery. The transition from in-person to live videoconferencing delivery was done based on feedback from patients about the burden of traveling for weekly visits in order to increase feasibility and to extend our reach to patients across the United States and internationally. In our pilot RCT [11], we showed that 3RP-NF is highly feasible and acceptable when delivered via live videoconferencing. Moreover, we showed that participation in 3RP-NF resulted in greater sustained improvement in QoL, psychosocial functioning [11], and resiliency variables [13] compared with an active control program (the Health Enhancement Program for Neurofibromatosis [HEP-NF]) [14], which was also delivered via videoconferencing using a group format.

We are now conducting the first fully powered efficacy RCT in adults with NF (N=224). The primary aim of this study is to determine the efficacy and durability of 3RP-NF versus HEP-NF regarding the coprimary outcomes of physical health QoL and psychological health QoL. Secondary outcomes include social relationship QoL, environment QoL, and psychosocial and resiliency measures. We hypothesize that 3RP-NF will be more efficacious compared with HEP-NF in improving coprimary and secondary outcomes from baseline to the end of active training and that the benefits of 3RP-NF participation will be maintained at 6- and 12-month follow-ups. The secondary aim is to examine the degree to which treatment-dependent changes in the coprimary outcomes are mediated by improvements in depression, anxiety, pain intensity, pain interference, social support, gratitude, optimism, mindfulness, empathy, coping ability, and stress (conceptual mediators) and modified by NF type, age, race, ethnicity, learning disability, and education level. We also plan to ascertain the minimal clinically important difference (MCID) for QoL variables for NF from baseline to posttraining, baseline to 6 months, and baseline to 12 months. This paper describes the study protocol.

## Methods

### Study Design

**Figure 1:**
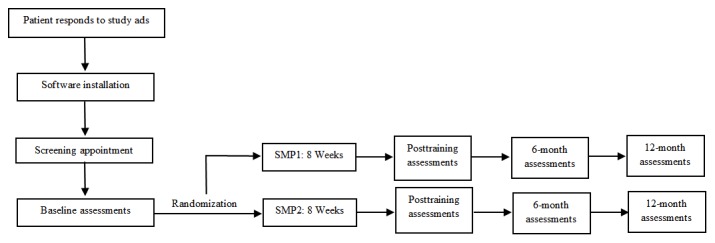
Study design. SMP: stress management program.

This is an ongoing, single-blind RCT of the efficacy and durability of 3RP-NF versus HEP-NF in improving QoL and psychosocial functioning in adults with NF1, NF2, and Schwannomatosis. Both programs are delivered via secure live videoconferencing, which allows enrollment of English-speaking participants across the United States and internationally. To maintain the single-blind design, we refer to the 3RP-NF and HEP-NF as Stress Management Programs 1 and 2 (SMP1 and SMP2, respectively) in all study materials. We began enrolling participants in September 2017. A flowchart of the study design is presented in Figure 1.

**Study inclusion criteria.**

Participants fulfilling the following requirements are included:

- Those with a diagnosis of neurofibromatosis (NF)1, NF2, or Schwannomatosis
- Those aged ≥18 years
- Those capable of completing and fully understanding the informed consent process, study procedures, and assessments in English
- Those with at least a 6th grade self-reported reading level
- Those with self-reported difficulties coping with NF symptoms
- Those scoring 6 or higher on the Perceived Stress Scale 4-item

**Study exclusion criteria.**

Participants in any of the following categories are excluded:

- Those with a major medical comorbidity, which is not related to neurofibromatosis (NF) and expected to worsen within the next 12 months
- Those with a recent change in antidepressant medication (within the past 3 months)
- Those with recent participation in cognitive behavioral or relaxation therapy (within the past 3 months)
- Those with a significant mental health diagnosis requiring immediate treatment (eg, untreated bipolar disorder, psychotic disorder, and active substance dependence) obtained via self-report and observation during prescreening
- Those unable or unwilling to complete assessments electronically via Research Electronic Data Capture
- Those unable or unwilling to participate in group videoconferencing sessions

### Setting

This study is being conducted in a large academic medical center in the northeast region of the United States. The use of virtual recruitment and live videoconferencing for intervention delivery allows participants from the United States and internationally to engage in the study from the comfort of their own homes or any location with internet access. Participants are recruited through the NF Registry at the Children’s Tumor Foundation (CTF), which has over 7000 members in the United States and internationally, various NF groups and clinics across the United States, and international NF centers (eg, England and Australia).

### Inclusion and Exclusion Criteria

The inclusion and exclusion criteria are detailed in Textboxes 1 and 2, respectively. The criteria were selected based on the guidelines for psychosocial treatment development [15] and for bridging efficacy with effectiveness [16]. These criteria were meant to be as inclusive as possible to maximize reach and uptake while reducing potential study confounders. Consistent with prior research that has utilized the Perceived Stress Scale 4-item[17] (PSS-4) measure as a screening tool for elevated stress, the potential participants are required to score at least 6 on PSS-4 [18,19]. Screening is conducted via secure live videoconferencing.

### Recruitment

Our institutional review board (IRB)-approved study advertisement is distributed electronically (eg, email listserv) and on paper through the CTF registry, national and international NF organizations, NF social media groups, and at NF clinics across the world. In addition, an IRB-approved recruitment video with snippets of patients’ experiences with study participation was created and disseminated at NF patient forums and on the official website of CTF. Potential participants send emails to our study coordinator who responds within 24 hours using a scripted email describing the study and offering the potential participants the opportunity to schedule a live videoconference screening with study staff. The study coordinator attaches the informed consent to the email to provide individuals time to review the study procedures, but instructs the potential participants not to sign the consent form before the screening appointment. The potential participants are provided 2 days after the initial email contact to respond before the study coordinator sends a follow-up email. The study coordinator stops contacting the potential participants after three unanswered emails.

### Vidyo Software Installation

All study appointments are conducted using the secure, Health Insurance Portability and Accountability Act (HIPAA)-approved live videoconferencing software Vidyo, which is a user-friendly platform used clinically at our academic medical center. Prior to the screening appointment, potential participants receive instructions via email to download, install, and access Vidyo on their personal, webcam-equipped, internet-connected devices (eg, laptop, desktop computers, and tablets). Participants are informed that devices with screens that are as large as possible are preferred to provide them with the best video-viewing experience. However, to increase generalizability and reach, participants who do not own compatible computers or tablets are allowed to use smartphones if necessary. The study coordinator also offers telephone appointments to assist potential participants with software installation and configuration (eg, ensuring the software is granted access to microphones and webcams) as needed.

### Technical Considerations

Participants have different levels of experience using technology and many have NF-related learning disabilities. These issues present several challenges in installing and configuring the videoconferencing software. First, when using a tablet or smartphone, the software must be downloaded as an “app” acquired through an app distribution platform (eg, app store or Google play store), which requires the user to have and access their own accounts. Users who are unfamiliar with “app stores” may require assistance creating or accessing an account and initiating an app download and installation. Second, the Vidyo software is designed to autodetect speakers, microphones, and webcams installed on the device. However, in instances where the device has multiple forms of hardware installed or where autodetection is unsuccessful, the study coordinator may need to assist patients in software configuration. Third, different types of operating systems (eg, Mac OS X, Microsoft Windows) have slight variations regarding the installation and configuration process as well as the user interface. The study coordinator has undergone extensive training to be able to assist participants with various technical challenges to ensure that all participants are provided the opportunity to enroll regardless of their prior experience using technology. The coordinator has also received specific training on how to interact with potential participants who might feel intimidated by the technological aspect of the project to make them feel comfortable and at ease. Furthermore, the team has prepared simple, easy-to-use, step-by-step instructions that are distributed to participants.

### Screening and Enrollment

After the potential participants have successfully installed Vidyo, the study coordinator schedules a virtual screening appointment during which the study staff determines eligibility and performs the informed consent process. Eligibility is determined based on the inclusion and exclusion criteria presented in Textboxes 1 and 2, respectively. All eligible participants are provided with an overview of the study procedures. The study therapist ensures that potential participants have a thorough understanding of the study procedures and are comfortable with the program format. The potential participants are encouraged to ask any questions about the informed consent document and study procedures, and the study therapist answers all study-related questions before informed consent is obtained. Eligible individuals who agree to participate are scheduled for the next available group (stratified by NF type) and are provided with instructions for returning the signed consent form to the study coordinator electronically (ie, via email or fax). Our IRB considers participants to be enrolled in the study when they return the informed consent document.

### Scheduling Considerations

The use of secure live videoconferencing allows participants to access the group session wherever they have a reliable internet connection and privacy. Thus, both participants and study therapists have flexibility in scheduling by having the option to access the treatment from home or office if privacy and internet needs are met. The ability to enroll geographically diverse participants is a strength of the current protocol. However, geographic diversity, which spans time zones across the globe, and an individual randomization schedule (see the subsequent section on randomization) pose challenges to scheduling that are addressed as follows: First, each pair of treatment groups (ie, 3RP-NF and HEP-NF) is facilitated consecutively within a 3-hour window (ie, 90 minutes per group) by a single study therapist; please see the “Randomization Considerations” section for details. Participant availability is required for the full 3-hour window to ensure that the participant is able to attend either group to which he or she is randomized. Second, we assess a wide range of participant availability, including during nights and weekends, to find a common time that will accommodate the study therapist’s schedule and as many participants as possible.

### Assignment to Treatment Arm

Participants are randomly assigned in a 1:1 ratio to either the 3RP-NF or the HEP-NF group via a permuted-block randomization (random blocks of 2 and 4), stratified by diagnosis (NF1, NF2, or Schwannomatosis), using a computer-generated (ie, SAS version 9.4; SAS Institute Inc, Cary, NC, USA) randomization schedule developed by our biostatistician. Randomization occurs individually for each participant after baseline assessments have been completed. After randomization, the study coordinator emails each participant to inform them of their assigned group and confirms when their group will meet within the original 3-hour window (ie, within the first 90 minutes or the last 90 minutes). To maintain blinding, participants are simply told whether they have been randomly assigned to SMP1 or SMP2. The study is single-blinded; thus, only participants are concealed of their group allocation.

### Randomization Considerations

Multiple randomization methods are available to assign participants to treatment arms within an RCT, including individual and cluster randomizations. We considered a cluster randomized design to facilitate scheduling feasibility, requiring participants to be available for only a 90-minute window and then randomizing that time slot to a treatment condition. However, given that our unit of inference is at the individual level and that cluster randomization can reduce statistical power [20], we selected an individual randomization schedule while considering the scheduling needs of participants, such as evening group times to accommodate working individuals. We also considered block randomization that would allow us to assign even numbers of participants to each pair of treatment groups. The current permuted-block design has considerable statistical and experimental design advantages that prevent the study team from deducing the next possible assignment. Due to limitations in the Vidyo software, we are unable to accommodate more than 8 participants in a single group. As we assign participants to a 3-hour block and then randomize them to treatment condition, we could ensure equal groups (ie, 8 per group) by using blocks of 16 for each time slot. However, that would allow the study team to deduce the final allocation before randomization. Thus, we decided to randomize patients in a permuted-block design, accepting the potential for uneven groups. Once a group has reached 8 participants, randomization for that full 3-hour time block is closed so as not to risk randomizing a 9th participant to a full group. While this can result in uneven groups (eg, 8 participants in one group and 6 in another), the integrity of the randomization schedule is maintained, and it has been feasible in our design to date.

### Treatment Conditions

Both treatment conditions include 8 weekly 90-minute group sessions delivered via secure live videoconferencing. Sessions are delivered on the same day and time each week for 8 consecutive weeks. Both treatments follow respective patient manuals, which are emailed as PDF documents to participants 2 days prior to their first session. The patient manuals are designed for a 6th grade reading and comprehension level. To further accommodate learning disabilities or other cognitive difficulties, participants are asked to bring the manual with them during each treatment session, either electronically or in print, to follow along and take notes during the session. A paper copy is mailed to participants who are not comfortable using the manual electronically and are unable to print the manual.

Each week, participants receive a reminder email on the day of their group session with instructions for logging into the Vidyo software. They are asked to email or call the study coordinator if they experience any technical difficulties and to email their study therapist if they are not able to attend the session. Moreover, participants who do not log into the session within 15 minutes of the start time receive a phone call from the study coordinator to inquire whether they are having technical difficulties and whether they will be able to attend the session. The study coordinator is available for the duration of each group session, and in the event of technical difficulties, he or she assists participants in real-time troubleshooting with the goal of enabling participants to log into the session as quickly as possible.

### Relaxation Response Resiliency Program for Neurofibromatosis

The 3RP-NF is a comprehensive, multimodal treatment program designed to improve the ability to cope with NF symptoms and stress. The program retains the main components and structure of the parent program, which combines the elicitation of the relaxation response (RR) with cognitive behavioral theory and the evolving field of positive psychology [12,21-28]. 3RP has three core components: (1) RR elicitation strategies to help decrease the stress response associated with NF symptoms and general life stress (eg, mindfulness skills, meditations, guided imagery, body scan); (2) stress and medical symptom appraisal and coping to help patients understand the interrelations among thoughts, behaviors, feelings, and sensations and learn adapting coping skills (eg, cognitive restructuring, problem solving, activity scheduling); and (3) growth enhancement or positive psychology skills that help patients experience pleasure and gratitude and engage in prosocial and empathic behaviors (eg, appreciations, use of humor, empathic communication). All skills have been modified to specifically address NF concerns identified through focus group discussions and exit interviews (ie, semistructured interviews conducted by the study therapist after participants completed the program) during 3RP-NF development process. Skills are taught using NF examples. The program also provides educational information on nutrition, exercise, and sleep hygiene.

Videoconferencing sessions consist of the study therapist and up to 8 participants, who can see each other but can block their video if desired. Each session begins with setting an agenda and a review of the material from all previous sessions. Each session introduces at least one RR skill and an additional cognitive behavioral or positive psychology skill, with presentation of NF-specific examples. The study therapist leads in-session exercises to demonstrate each skill and assigns home practice of the skills to facilitate mastery. In-session practice and review of all the skills taught in prior sessions is unique to the 3RP-NF and done to compensate for the high rates of cognitive and learning disabilities in the NF population. Before each session, participants receive an MP3 file recording with the particular RR skill that will be taught and practiced during the particular group session to aid with home practice. Between sessions, participants are asked to practice the skills daily, complete a home practice log, and email it to the study therapist at least 2 hours before the start of each group session to provide enough time for the study therapist to review each participant’s practice. Home practice includes three core elements: (1) setting 1 weekly goal; (2) practicing an RR skill, starting with 5 minutes daily for the first week and gradually increasing the length, frequency, or both of practice throughout the 8-week program; and (3) writing down 1-3 appreciation statements daily. Each session includes a review of home practice, including problem solving of barriers as needed, with specific feedback from patients. A complete description of treatment content by session is presented in Table 1.

**Table 1:** Outline of the Relaxation Response Resiliency Program for Neurofibromatosis.

Session	Topics	Skills
Symptom management, stress management, and resiliency training	Stress response Relaxation response Resiliency Mind-body connection	Single-pointed focus meditation Energy battery Specific, Measurable, Attainable, Realistic, and Time-based (SMART) goals Gratitude and appreciations
The relaxation response	Developing routines for consistent skill practice Recuperative sleep Relaxation response and emotions	Body scan Diaphragmatic breathing Sleep hygiene Identifying emotions and physical sensations
Stress and symptom awareness for patients with neurofibromatosis (NF)	Mindful awareness Stress awareness Social support	Mindful awareness meditations (eg, mindful eating) Identifying stress warning signals The social support diagram
Mending the mind and body of patients with NF	Movement to elicit the relaxation response Negative automatic thoughts (NATS) Thought distortions	Stretching and chair yoga for relaxation Adaptive thinking: identifying NATS and thought distortions Pleasant experiences to build resiliency
Creating an adaptive perspective	Adaptive thinking Healthy eating	Generating adaptive thoughts through reframing, use of positive emotions or beliefs, and acceptance Stop, breathe, reflect, choose Guided imagery Food pyramid
Promoting positivity	Positive psychology Optimistic explanatory style Physical activity	Loving kindness meditation Telling our stories with optimism Identifying relaxation signals Guidelines for health activity levels
Healing states of the mind	Empathy toward self and others Choosing appropriate coping strategies Acceptance	Contemplation Problem-solving and acceptance strategies Coping decision tree Mindful awareness Letter to self
Humor, empathy, and staying resilient	Staying resilient Humor	Idealized self-imagery Laughter in daily life SMART goals for continued skill practice

### The Health Enhancement Program for Neurofibromatosis

The parent Health Enhancement Program (HEP) [14] is a group-based health education program that addresses multiple domains of healthy living that are known to impact stress management, including sleep, diet, and physical activity. The HEP-NF has been adapted to address the specific needs of patients with NF, including educational information on NF-specific stressors and medical symptoms, NF-specific barriers to healthy lifestyle behaviors (eg, appearance concerns, pain), self-management of medical care, and navigating the medical system. Adaptations were made using educational materials from the CTF website, research literature on NF, and information from focus group discussions. The program does not teach any of the 3RP-NF skills.

As with the 3RP-NF sessions, each HEP-NF session is composed of the study therapist and up to 8 participants who are present in a shared videoconferencing meeting. Each session of the HEP-NF begins with setting an agenda and review of previous material. The study therapist presents educational information on the session topic and invites participants to provide examples from their own experiences. Participants are encouraged to pick a lifestyle skill learned in-session for use between sessions. A complete outline of content by session is presented in Table 2.

### Treatment Fidelity

We use guidelines established by the NIH Behavior Change Consortium to monitor 5 areas of treatment fidelity:

- Design
- Training
- Treatment delivery
- Receipt of treatment
- Enactment of treatment skills

**Table 2:** Outline of the Health Enhancement Program for Neurofibromatosis.

Session		Topics	Skills
Connection between physical and mental health		Stress and mental health Stress and physical illness Connection between lifestyle behaviors and physical and emotional health	Knowledge of lifestyle behaviors Goal setting
Neurofibromatosis (NF) and stress		Types of NF Stress associated with each NF type	Identifying personal NF stressors Identifying shared stressors among NF types Goal setting
Sleep and wellness		Sleep and physical and emotional well-being Sleep system (eg, circadian rhythm) Behavioral patterns and sleep	Sleep hygiene Stimulus control Checklist for better sleep
Exercise and wellness		Physical activity recommendations Maintaining a healthy weight	Identifying current activity patterns Identifying and problem solving barriers to activity Goal setting
**Nutrition**			
	Basic information	Food groups and portion sizes Calories and nutrient density	Reading and understanding nutrition labels My healthy plate guidelines Visualizing portion size
	Healthy weight and weight loss	Healthier meals and snacks Eating out healthy Weight and health Healthy weight loss	Preparing a shopping list Tips for eating out Calculating body mass index
Managing health care		Communicating with health care providers Preparing for a medical visit Medication adherence	Maintaining up to date records (eg, lists of doctors, medications, recent tests) Health diaries Role plays of preparing and asking questions of medical providers Tips for managing medications
Review		Review of healthy sleep Review of physical activity Review of nutrition Review of NF and stress Review of health care management	N/A^a^

^a^N/A: not applicable.

#### Design

All study staff attends weekly team meetings to monitor and record participant progression through the study, including treatment “dose” (eg, session attendance, out-of-session contact with therapists) and any deviations from the prescribed dose. In addition, the study coordinator and project director meet individually each week to review progress and upcoming tasks. The principal investigator (PI) participates in weekly meetings and conducts quality control checks on a monthly basis.

#### Training

All study therapists are advanced graduate students or PhD-level clinical psychologists with experience in mind-body therapy. Each study therapist attends the 8-week parent program 3RP, led by a seasoned clinician at our academic medical center, as a participant observer. The study therapists also undergo study-specific training including learning general information about NF; watching videos of patients with NF1, NF2, or Schwannomatosis who are describing their symptoms; and specific training on the adaptation and delivery of skills to patients with NF. The therapists receive training about the importance of adherence to the treatment manual and completion of study-related forms. The study therapists attend weekly in-person group supervision to assure adherence to the protocol, discussion of specific patient concerns (eg, home practice challenges), and review of upcoming group sessions.

#### Treatment Delivery

All study therapists deliver session content according to the respective patient manuals and complete adherence checklists after each session. The adherence checklists are reviewed by the PI weekly during clinical supervision. Furthermore, all study sessions are audiorecorded, and 15% are randomly selected to be reviewed for adherence.

#### Receipt of Treatment

The study therapists monitor and support patients’ ability to comprehend and utilize treatment by reviewing previous content and setting an agenda at the start of each session. The study therapists elicit feedback from patients about their comprehension, goals, motivation, and use of skills throughout each session as new material is taught. Participants are instructed to follow along and take notes in their treatment manual, and to review the manual out of session, to facilitate comprehension.

#### Enactment of Treatment Skills

Participants are instructed to set weekly goals for applying skills and information presented in each session. The study therapists review home practice and problem solve barriers weekly.

### Considerations for Participant Safety During a Virtually Delivered Program

The safety of participants is evaluated at multiple study points. At the time of enrollment, participants are asked to provide the names and phone numbers of two family members or friends who could be contacted in case of emergency or if study staff has concerns about a participant’s safety (eg, inability to contact participant following endorsement of suicidal ideation). At baseline, participants complete a measure of depressive symptomatology, which asks about the frequency of “Thoughts that you would be better off dead or of hurting yourself in some way.” If participants provide any endorsement (ie, any response greater than “not at all”) to this item, the electronic data capture system automatically emails the study coordinator, project director, and PI. The study coordinator and project director communicate with the PI to provide patient contact and emergency contact information if needed. The PI, a licensed clinical psychologist, immediately contacts the participant via phone to conduct a suicide risk assessment, including the development of a safety plan and determination of need for higher level of care. If the PI is unable to contact the study participant within 24 hours, the PI then calls the patient’s individual emergency contacts to locate the participant and assess safety. The safety of participants is always prioritized over study participation. Participants who are determined to need a higher level of care or refuse to comply with safety procedures (eg, refuse to conduct a risk assessment over the telephone) are removed from the study and provided with information about resources for care as appropriate. Participants who are determined to be at low suicide risk and appropriate for continuation in the study are monitored by the study therapists and discussed during weekly clinical supervision. At follow-up, the same procedures for risk assessment and referral to higher levels of care, as needed, are followed.

### Assessments

Participants complete assessments online through the secure Web app, Research Electronic Data Capture (REDCap) [29]. Baseline assessments are completed after obtaining informed consent, prior to randomization, and no more than 2 weeks prior to the first group session. Posttraining assessments are emailed to participants within 24 hours of the final group session. Participants who have not completed the posttraining assessments within 3 days receive a reminder email from the study coordinator and a phone call from the study therapist. The study therapists contacts the remaining participants daily (via phone or email as appropriate) to facilitate the completion of assessments within 1 week of the final group session. Furthermore, 6- and 12-month follow-up surveys are emailed to participants 1 week before the respective assessment due date. Participants who do not complete the questionnaires within that week then receive three additional email reminders and up to three phone calls from the study therapist or PI. The study staff ceases attempt to obtain 6-month follow-up after 2 months (ie, month 8). Participants who do not complete the 6-month follow-up are contacted as usual to complete the 12-month assessment. Participants who do not complete the 12-month follow-up after 2 months (ie, month 14) are considered lost to follow-up, and their participation in the study is terminated. Each assessment is completed at each time point unless otherwise specified.

#### Sociodemographic Information

Gender, age, race, ethnicity, marital status, NF type, presence of a learning disability (self-report), and education level (number of years in school) are collected using a demographic questionnaire. This assessment is only delivered at baseline.

#### Primary Outcomes: Physical Health Quality of Life and Psychological Health Quality of Life

The World Health Organization Quality of Life Brief version (WHOQOL-BREF) [30] is a 26-item self-report survey used to measure four domains of QoL: physical health (7 items), psychological health (6 items), social relationships (3 items), and environmental health (8 items). The physical health QoL and psychological health QoL domains are the coprimary outcomes of this study. The physical health domain assesses an individual’s ability to participate in activities of daily living and his or her dependence on medicinal treatments and medical aids for daily functioning, energy and fatigue, mobility, pain and discomfort, sleep and rest, and work capacity. The psychological health domain assesses satisfaction with bodily image, frequency of negative and positive emotions, self-esteem, spirituality, and ability to concentrate. Scores are reported as transformed domain scores (0-100), with high scores depicting a greater QoL. No NF-specific MCID has been established for the WHOQOL-BREF; however, a 6.25-unit improvement has been extrapolated from the MCID for patients with cancer available for the parent scale WHOQOL-100. Thus, a 6.25-unit increase is used as an indicator of clinically meaningful improvement in physical health QoL and psychological health QoL in the current study.

#### Secondary Outcomes: Social Relationship Quality of Life and Environment Quality of Life

The social relationship QoL and environment QoL domains of the WHOQOL-BREF are secondary outcomes of this study. The social relationship domain assesses satisfaction with personal relationships, availability of social support, and satisfaction with sexual relationships. The environmental health domain assesses perceived financial resources, physical safety and security, accessibility and quality of health care and social services, home environment, opportunities for learning and growth, opportunities for recreation and leisure activities, physical environment (pollution, noise, climate, traffic), and transportation. The domains are scored in accordance with the instructions mentioned above.

#### Conceptual Mediators

##### Depression

The Patient Health Questionnaire 9-Item version [31] is a self-report survey used to measure the frequency of depression symptoms (eg, little interest or pleasure in doing things, trouble falling or staying asleep, poor appetite or overeating, trouble concentrating) over the past 2 weeks. Responses are formatted as a 4-point Likert scale ranging from 0 (“Not at All”) to 3 (“Nearly Every Day”). The items are summed to generate a total score, with higher scores indicating greater severity of depression symptoms.

##### Anxiety

The Generalized Anxiety Disorder 7-Item version [32] is a self-report survey used to measure the frequency of anxiety symptoms (eg, feeling nervous or on edge, not being able to stop or control worrying, being restless, becoming easily annoyed or irritable) over the last 2 weeks. Responses are formatted as a 4-point Likert scale ranging from 0 (“Not at All”) to 3 (“Nearly Every Day”). The items are summed to generate a total score, and higher scores indicate greater severity of anxiety symptoms.

##### Pain Intensity

The characteristic pain intensity subscale of the Graded Chronic Pain Scale [33] uses three separate 11-point numerical rating scales (“0 = no pain” to “10 = pain as bad as it could be”) to assess current momentary pain, worst pain, and average pain over the previous week.

##### Pain Interference

The PROMIS Pain Interference version Short Form 8a [34] is an 8-item self-report survey used to measure the extent to which pain interferes with activities of daily living, including household chores, work, and social activities, over the past 7 days. Responses are formatted as a 5-point Likert scale ranging from 1 (“Not at All”) to 5 (“Very Much”). The items are summed to generate a total score and cross-referenced with the score conversion table to translate the raw score to a T-score for each participant. The T-score rescales the raw score into a standardized score with a mean of 50 and a SD of 10. Therefore, an individual with a T-score of 40 is 1 SD below the mean.

##### Stress

The Perceived Stress Scale 10-Item version [35,36] is a self-report survey that assesses the frequency of thoughts or feelings related to stress (eg, becoming upset with something unexpected, feeling unable to control important things in life, and feeling incapable of coping with things to do) within the past month. Responses are formatted as a 5-point Likert scale ranging from 0 (“Never”) to 4 (“Very Often”). Negatively worded items (4, 5, 7, and 8) are reverse scored, and then, all items are summed to generate a total score, with higher scores indicating greater perceived stress.

##### Social Support

The Medical Outcome Study Social Support Survey [37] is a 19-item self-report survey used to measure perceived social support. The survey asks how often the different kinds of support are available to the respondent as needed, divided into three domains: emotional or informational support, affectionate support, and positive social interaction. Responses are formatted as a 5-point Likert scale ranging from 1 (“None of the Time”) to 5 (“All of the Time”). The items are averaged, with higher scores indicating greater availability of social support.

##### Gratitude

The Gratitude Questionnaire 6-Item version [38] is a self-report survey used to measure a general tendency to experience gratitude (eg, being appreciative of people, events, and situations). Responses are formatted as a 7-point Likert scale ranging from 1 (“Strongly Disagree”) to 7 (“Strongly Agree”). Negatively worded items are reverse scored, and then, all items are summed to generate a total score, with higher scores indicating greater gratitude.

##### Optimism

The Life Orientation Test Revised [39] is an 11-item self-report survey used to measure a tendency toward optimism (ie, expecting the best in uncertainty and expectations on whether good or bad things will happen). Responses are formatted as a 5-point Likert scale ranging from 0 (“Strongly Disagree”) to 4 (“Strongly Agree”). Negatively worded items are reverse scored, and then, all items are summed to generate a total score, with higher scores indicating greater optimism.

##### Coping Ability

The Measure of Current Status Part A [40] is a 13-item self-report survey used to assess perceived ability to cope (eg, capability to use coping techniques, being able to recognize stress). Responses are formatted as a 5-point Likert scale ranging from 0 (“I Cannot Do This At All”) to 4 (“I Can Do This Extremely Well”). The measure yields four subscales: relaxation, awareness of tension, assertiveness, and coping confidence. Items pertaining to each subscale are summed, with higher scores on each subscale indicating greater ability to cope in each respective manner.

##### Mindfulness

The Cognitive and Affective Mindfulness Revised [41] scale is a 12-item self-report survey used to measure mindfulness (ie, the ability to pay attention to the present moment in a nonjudgmental manner). The survey asks the respondent to indicate how often they related to their thoughts and feelings mindfully (eg, focused on the present moment, ability to concentrate). Responses are formatted as a 4-point Likert scale ranging from 1 (“Rarely or Not at All”) to 4 (“Almost Always”). Negatively worded items are reverse scored, and then, all items are summed to generate a total score, with higher scores representing greater mindfulness.

##### Empathy

The 7-item empathic concern subscale of the Interpersonal Reactivity Index [42] is a self-report survey used to measure empathy (ie, feeling concerned for others, being protective of others, and being sensitive to others). Responses are formatted as a 5-point Likert scale ranging from 1 (“Does Not Describe Me Well”) to 5 (“Describes Me Very Well”). Items are summed to generate a total score, with higher scores indicating greater empathy.

##### Perceived Improvement

The Patient Perception of Improvement (PPI) [43] is a single self-report item that asks the question, “Do you think that you are now better, about the same or worse as compared to before the intervention?” The item was modified for our study to assess perceived improvement in QoL by asking, “Do you think your quality of life is now better, about the same or worse as compared to before the intervention?” Response options are “Substantially worse,” “Minimally worse,” “About the same,” “Minimally better,” and “Substantially better.” Separate items are used for each QoL domain (physical health, psychological health, social relationships, and environmental health). This assessment is delivered at posttraining and at 6- and 12-month follow-ups.

### Data Analysis

#### Power Analysis

SAS version 9.4 was used to calculate the power analysis. The effective SDs for the change from baseline to posttraining in physical health and psychological health QoL based on a repeated-measures analysis of variance (ANOVA) of our preliminary data [11] were 14.7 and 10.4 units, respectively. The effective SDs from posttraining to 6-month follow-up were 11.4 and 10.0 units, respectively. Based on these estimates, assuming an MCID of 6.25 units, allowing up to 5% loss to follow-up by posttraining assessment, and testing each of the coprimary outcomes at *P*<.03 (two-tailed), a total of 224 participants will afford 80% power for physical health-related QoL and 96% power for psychological health QoL. Allowing up to 20% loss to follow-up by the 6-month assessment, the study will have 99% power to declare noninferiority of 3RP-NF versus HEP-NF if the true treatment-dependent difference in the maintenance of any change from baseline to posttraining is zero.

#### Primary and Secondary Outcomes

SAS version 9.4 will be used for the statistical analyses. Treatment effects on primary and secondary outcomes will be analyzed using a shared-baseline, linear mixed model with fully unstructured covariance among up to four repeated measures (baseline, posttraining, and 6- and 12-month follow-ups). The mixed model uses all available data. Participants with missed assessments and those lost to follow-up are included in the analysis. The estimated covariance among repeated measures implicitly imputes missing data. Thus, the model yields unbiased estimates if any missing data are predictable from the observed assessments. Compliance with the intervention and attendance during the group sessions specifically will not be included in the analyses of treatment effect. It is impossible to deduce whether any observed association between outcomes and attendance reflects the effects of increased training on the outcomes or effects of differential outcomes on attendance. It is equally plausible that participants experiencing better outcomes are more likely to attend training sessions as attending additional training sessions improves outcomes. The shared-baseline assumption reflects the true state of the population prior to randomization, and it has the benefit of adjusting for chance differences at baseline [44]. For each outcome, we will compare the effect of 3RP-NF versus HEP-NF on changes from baseline to posttraining and to 6- and 12-month follow-up times using linear contrasts and will report point estimates and their 95% CIs. The two coprimary outcomes will be declared significant for *P*<.05 two-tailed. Persistence of a benefit from 3RP-NF from posttraining to 6- and 12-month follow-up times will be analyzed as a noninferiority test of durability. Noninferiority of 3RP-NF in maintaining benefits relative to HEP-NF will be declared if the lower one-sided 95% confidence bound for a given coprimary outcome is less than 6.25 units (the estimated MCID) in favor of HEP-NF. Several sensitivity analyses will be explored using alternative models. Changes in the scores calculated from baseline to posttraining and to 6- and 12-months will be separately analyzed using Wilcoxon rank sum test to avoid any parametric assumptions about the data. More parsimonious covariance structures will be considered using random participant-specific intercepts, slopes, and quadratic terms (ie, growth curve analysis). Baseline parameters such as NRS pain and their interactions with visit will be included to account for chance differences due to randomization and to explain sources of variation in responses that are independent of the treatment group. All randomized participants will be included in our primary efficacy analyses as randomized, following the intention-to-treat principle.

#### Mediation and Moderation Analyses

If the 3RP-NF intervention improves some or all of the coprimary and secondary outcomes compared with HEP-NF, we will explore the extent to which this relationship is mediated by psychosocial variables (eg, depression, anxiety, pain interference, and pain intensity). The degree to which a given psychosocial variable mediates the effect of 3RP-NF treatment on a given outcome will be estimated from the pure natural indirect effect from a causal model that includes potential interaction between the intervention and the mediator but assumes no unmeasured confounders [45]. Changes in scores from baseline to each follow-up assessment will be analyzed. Evidence of mediation will be inferred if the CI does not cover zero. The mediation effect size will be determined by the proportion of the total effect that is attributable to the mediation (ie, the mediated effect divided by total effect). This method is consistent with that of Baron and Kenny [44] and has been updated by Kraemer et al [46]; however, it extends the analysis by allowing us to test the significance of the mediated effect and quantify the magnitude of the mediation.

The possible effect of moderators of a beneficial effect of 3RP-NF will be investigated by adding a given moderator (eg, contrasting treatment response by NF1, NF2, and Schwannomatosis), moderator × treatment, and moderator × treatment × visit interaction terms to the repeated-measures ANOVA using methodology described for analyses of primary outcomes. The specific linear contrasts of the moderator × treatment × visit interaction terms will be used to test for differential 3RP-NF dependent benefit in improvements from baseline to posttraining and to 6- or 12-month follow-up that are a function of diagnosis, age, and race or ethnicity. While we have not designed the study to have a good power to detect differences based on NF type, we have optimized our power to detect differences based on NF type by stratifying randomization by diagnosis, given the available sample size and distribution of NF types.

We will develop WHOQOL-BREF MCID thresholds specific for NF using an anchor-based approach based on participants’ self-reports of important changes on the PPI. We will use mixed model cumulative logistic regression to model ordinal responses on the PPI. Each model will include fixed effects of physical health QoL or psychological health QoL and follow-up visit and random participant-specific intercepts to account for correlations among repeated measures. If the variable “visit” (ie, baseline, posttraining, and 6- and 12-month follow-ups) is not significant, it will be dropped from the model. The estimated MCIDs will be the ones that best discriminate participants who report being “About the same” versus “Minimally better” on the PPI, that is, the physical health- or psychological health-related QoL score for which the predicted probability of being “About the same” and “Minimally better” on the PPI is equal.

### Data Management

To maximize accuracy and security, all survey data are collected and stored on a secure and HIPAA-compliant Web-based REDCap [29] data system hosted by our academic medical center. Data are stored on password-protected computers that are kept at secure locations. Paper data files (with coded subject identification) are stored in a locked filing cabinet accessible only to the research team. A unique anonymous identifier is assigned to each subject; subsequently, all collected data are associated exclusively with this identifier. This includes all questionnaires administered over the course of the study as well as home practice logs.

## Results

The trial is ongoing. Thus far, we have recruited 55 patients and aim to recruit a total of 224. Recruitment will close in May 2020; we plan to complete data analyses by June 2021.

## Discussion

NF is a prevalent and incurable condition associated with decreased QoL and high psychosocial comorbidities. The current standard of care for NF is predominantly biomedical. Using a sequential approach and direct feedback from patients, we adapted an evidence-based mind-body program (the 3RP) for the specific needs of patients with NF (3RP-NF). In a pilot RCT, we showed that 3RP-NF has excellent feasibility and acceptability [11]. Moreover, we showed that participation in 3RP-NF was associated with sustained improvement in QoL, psychosocial functioning [11], and multiple dimensions of resiliency [13] (eg, perceived coping ability, perceived social support, and mindfulness) relative to an educational program tailored for the needs of patients with NF (HEP-NF). To remove barriers to care for this rare disease and to increase generalizability, both programs were delivered to patients with NF1, NF2, and Schwannomatosis across the United States and internationally using live videoconferencing.

This paper describes the study design and specific strategies used to conduct an innovative, fully powered RCT of 3RP-NF versus HEP-NF administered via live videoconferencing to adult patients with NF across the United States and internationally. We provide details on the benefits and challenges of delivering psychosocial care using secure live videoconferencing, procedures for keeping participants blinded throughout study participation, means of accommodating patients from different time zones, techniques for keeping patients engaged in treatment, and methods of monitoring and addressing the safety of participants. This information is invaluable for future trials using live videoconferencing, and it represents a novel model for delivery of care to patients with rare diseases or to those in remote areas.

Results of this trial will not only provide important information on the efficacy and durability of 3RP-NF versus HEP-NF over a year but also allow us to understand whether the specific targets of the 3RP-NF interventions—mindfulness, coping, social support, optimism, and others—are plausible mechanisms for improvements in the primary outcomes. We will also be able to address whether any benefits of 3RP-NF are dependent on demographic variables, NF type, or self-reported learning disability. Furthermore, using direct feedback from patients, we will be able to calculate NF-specific MCID scores for the physical health- and psychological health-related QoL measures, which will allow a patient-specific evaluation of improvement in the four domains of QoL.

Both the 3RP-NF and the HEP-NF have been adapted iteratively based on feedback from patients with NF and have showed excellent feasibility, acceptability, and preliminary efficacy in our prior work. If the current trial replicates our prior pilot study [11,13] and confirms our hypotheses that 3RP-NF is superior to HEP-NF in improving physical health QoL and psychological health QoL as well as other secondary outcomes, we aim to implement and disseminate 3RP-NF as part of standard of care through the major NF centers within the United States and internationally as well as through CTF. Using the CTF-sponsored annual clinic’s meeting and general support from CTF, we would aim to train a variety of providers (eg, psychologists, social workers, nurses, genetic counselors). The original 3RP has been delivered successfully in clinical practice at our institution by nonpsychologists.

Despite the novelty of this trial, there are several limitations. First, although we are using extensive national and international recruitment modalities, recruiting ethnically and racially diverse participants is challenging. Our pilot RCT [11] enrolled primarily white patients, and we have strategies in place to diversify our patient population. Specifically, we have a strong presence in the NF community and have developed a recruitment video that includes racially diverse patients with NF who positively describe their experiences within the study. Second, although we are recruiting international patients, we are delivering the program and assessments in English only. Patients who are not fluent in English are not able to enroll. If the results of this RCT show that the 3RP-NF is an efficacious program, future work will involve translation of 3RP-NF as part of international dissemination efforts. Furthermore, the majority of patients with NF1 enrolled in both our pilot study and current trial do not have severe cutaneous tumors, suggesting that these patients may not be comfortable participating in a group intervention due to appearance-related concerns. In addition, although we took extensive measures to keep participants blinded to HEP-NF and 3RP-NF, we risk unblinding participants who searched for our research [11,13].

In summary, this is the first psychosocial RCT delivered via live videoconferencing in patients with NF, and it provides valuable information about the design, structure, challenges, and benefits associated with this study design and delivery modality. Results will inform implementation efforts and future clinical trials in other NF populations (eg, adolescents with NF1 and NF2, parents of children with NF1 and NF2, and adults with NF2 who are deaf) as well as other trials targeting geographically diverse individuals with rare diseases. Moreover, our findings will potentially extend the applicability of the 3RP mind-body program core skills to other medical populations and increase our understanding of the mechanisms of its efficacy across medical populations.

## Appendix

Multimedia Appendix 1 Peer-review report from the Department of Defense office of the Congressionally Directed Medical Research Programs (CDMRP).

## Abbreviations

ANOVA: analysis of variance
CTF: Children’s Tumor Foundation
DOD: Department of Defense
HEP-NF: Health Enhancement Program for Neurofibromatosis
HIPAA: Health Insurance Portability and Accountability Act
IRB: institutional review board
MCID: minimal clinically important difference
NATS: negative automatic thoughts
NF: neurofibromatosis
PI: principal investigator
PPI-4: Perceived Stress Scale 4-item
PSS: Patient Perception of Improvement
QoL: quality of life
RCT: randomized controlled trial
RR: relaxation response
SMART: Specific, Measurable, Attainable, Realistic, and Time-based
SMP: Stress Management Programs
WHOQOL-BREF: World Health Organization Quality of Life Brief version
