# Supplementary material for: Mind-Body Treatment for International English-Speaking Adults With Neurofibromatosis via Live Videoconferencing: Protocol for a Single-Blind Randomized Controlled Trial
Source: JMIR Res Protoc. 2018 Oct 23;7(10):e11008. doi: 10.2196/11008 (PMC6231775; doi:10.2196/11008)
Supplement: Multimedia Appendix 1 [file resprot_v7i10e11008_app1.pdf]

Department of Defense
U.S. Army Medical Research and Materiel Command
Congressionally Directed Medical Research Programs
2016 Neurofibromatosis Research Program
Clinical Trial Award
Peer Review Summary Statement

CDMRP Log Number: NF160027
Grants.gov ID Number: GRANT12221721
Meeting Dates: 09/16/2016-09/16/2016
Review Panel: Clinical Trial - Clinical Consortium

Project Duration: 48 months
Total Budget Requested: $1,335,614
Direct Costs: $781,061
Indirect Costs: $554,553

Title: Resiliency Training for Adults With NF1, NF2, and Schwannomatosis: A Randomized Controlled Trial via Live Video
Principal Investigator: Ana-Maria Vranceanu
Performing Organization: Massachusetts General Hospital
Contracting Organization: Massachusetts General Hospital

Overview

The Principal Investigator (PI) of this application proposes to conduct a randomized controlled trial (RCT) to test the efficacy of the Relaxation Response Resiliency Program (3RP) for adult patients with neurofibromatosis (NF) (3RP-NF) as compared to the Health Enhancement Program for patients with NF (HEP-NF) in improving coprimary (physical and psychological quality of life [QoL] and secondary [social QoL, environmental QoL, depression, anxiety, pain intensity and pain interference]) outcomes. The project's specific aims are (1) to determine the efficacy of the mind-body 3RP-NF in comparison to an active-attention placebo control HEP-NF among patients with neurofibromatosis type1 (NF1), neurofibromatosis type 2 (NF2), and schwannomatosis; and (2) to examine the degree to which treatment-dependent improvements in the primary outcomes are mediated by improvements in depression, anxiety, pain, and pain interference (conceptual mediators) and modified by NF type, age, race/ethnicity, and learning disability (conceptual moderators). The study is a single-blind RCT, and the intervention is 3RP-NF. The projected outcomes are that 3RP-NF will be more efficacious in reducing primary and secondary outcomes at postintervention, and effects will be maintained at 6- and 12-month follow-ups, compared to HEP-NF. In addition, it is projected that treatment-dependent improvements in the mediators will explain benefits from 3RP-NF for the primary outcomes, and the moderators NF type, age, learning disability, and race/ethnicity will be associated with differential benefit from 3RP-NF for the primary outcomes.

Procurement Sensitive Document
Do not copy or distribute without CDMRP written permission.
Prepared by SRA International, Inc., a support contractor to CDMRP

	Average Score	Standard Deviation
**Overall Evaluation**<br>Rating Scale: 1.0 (highest merit) to 5.0 (lowest merit)	1.2<br>**(Outstanding)**	0.2
**Evaluation Criteria**<br>Rating Scale: 10.0 (highest merit) to 1.0 (lowest merit)	**Average Score**	
**Clinical Impact**	9.2	
**Intervention**	9.1	
**Research Strategy**	9.0	
**Recruitment, Accrual, and Feasibility**	8.9	
**Statistical Plan**	8.4	
**Transition Plan**	8.5	
**Personnel and Communication**	9.9	

SCORED EVALUATION CRITERIA

Clinical Impact
Average Score: 9.2

Scientist Reviewer A

Strengths: This study addresses a critical, current, understudied, and undertreated problem in NF patient care—physical and mental health/QoL. The application addresses the NFRP Health Disparity and Nontumor Manifestations Areas of Emphasis very well. The study will recruit individuals with NF1, NF2, and schwannomatosis, all populations of individuals who could and would likely benefit from the proposed intervention (pilot study results from this group have already identified this to have a positive impact on this group of individuals). There are short- and long-term benefits to this trial, particularly if the hypotheses are supported. There would be immediate and sustained benefits to the participants in the study with regard to QoL. More globally, the success of this study will open opportunities for future research on nonmedical/behavioral interventions for addressing the psychological needs of these individuals and potentially other areas of need (eg, cognition). This is particularly relevant as no drug to date has translated successfully from animal models to humans (cognitive). This intervention compares favorably with the current standard of care and may be an improvement over that standard in terms of acceptability and feasibility. Current similar interventions would be done face to face.

Weaknesses: A minor weakness is that there may be individuals who are either not comfortable with telemedicine approaches to treatment or may not have the technical abilities to successfully engage in this manner. Coverage for such services currently is highly variable, which relates to the comparison to current standard practices (face-to-face therapeutic services are commonly covered by insurance companies).

Procurement Sensitive Document
Do not copy or distribute without CDMRP written permission.

Scientist Reviewer B

Strengths: This study will evaluate the efficacy of a mind-body intervention program (3RP-NF) in improving quality of life of individuals with NF. The PI's previous work has demonstrated both the short- and long-term benefits of the program for individuals with NF. The PI has carefully considered how to represent the larger population of patients with NF in her application by not only including individuals with diagnoses NF1, NF2, and schwannomatosis, but also by completing the intervention via a Web-based system, allowing individuals across the country to participate. Previous studies, as well as the PI's own focus groups, have identified QoL, anxiety, depression, and pain to be significant concerns of individuals with NF, to the point that they feel these symptoms have been "ignored" by research. As such, this study will have great relevance for individuals with NF. Within the field of NF, this type of intervention study is innovative and incredibly needed. Unlike other disease groups, where psychosocial interventions have been researched and are routinely provided as standard of care, this has not been done within NF. As other chronic illness groups have found psychosocial interventions to be an important aspect of treatment, not having empirically proven interventions for individuals with NF represents a huge gap in NF research and impacts the quality of care given to these individuals. As such, this study has the possibility of having great impact on how individuals with NF are supported and treated.

Weaknesses: No weaknesses were noted.

Consumer Reviewer

Strengths: FY16 NFRP areas of emphasis addressed are health disparity with the lack of psychosocial treatments for patients with NF1, NF2, and schwannomatosis and within nontumor manifestation. It addresses QoL, pain, and other psychosocial variables through innovative health care delivery systems and the use of technology. Research findings show patients with NF, all types, have more symptoms of depression, pain, and anxiety, lower self-esteem, and lower QoL than general population norms. These psychosocial problems have not been addressed. This RCT will test the 3RP-NF mind-body program with a control intervention, HEP-NF, that provides only educational information and no coping skills. The participants will be 224 adults with NF1, NF2, and schwannomatosis. Recruitment will be in the United States and Canada. Children's Tumor Foundation (CTF) will assist with their patient registry ensuring generalizability of results. Short-term positive results would support dissemination of the 3RP-NF and the contention that the 3RP-NF is durable and more capable of producing results of improved physical health and improved psychological QoL. The long-term goal is to make mind-body care available to all patients with NF. The importance of psychosocial aspects of NF1, NF2, and schwannomatosis cannot be overlooked. As with the care of all chronic diseases such as cancer and heart disease, patient care has transitioned from all biomedical to biopsychosocial, where mind-body treatments are intertwined within medical care. Research data have shown that such psychosocial models have shown that QoL has improved and also enhanced outcomes of medical modalities. The benefits to all patients with NF would increase with this significant application.

Weaknesses: No weaknesses were noted.

Intervention
Average Score: 9.1

Procurement Sensitive Document
Do not copy or distribute without CDMRP written permission.

Scientist Reviewer A

Strengths: The PI presents clear evidence of support and availability for the intervention. The PI and study team have done pilot studies with this intervention and also use it clinically in practice. There is clear technological support and institutional support ensuring the availability of this intervention for the duration of the trial. This intervention very much addresses clinical needs in NF and in a manner that widens the availability to patients who otherwise may not be able to access such an intervention (telemedicine). Standard therapies are available clinically, and there are increasing numbers of telemedicine services available (although insurance coverage varies widely). There is evidence that the proposed intervention and modality are at least commensurate with currently available (in-person) interventions and standards of care. Outcome questionnaires and measures are appropriate and well described in the application. The PI has addressed any concerns about confidentiality as they are using technology that is HIPAA compliant.

Weaknesses: No weaknesses were noted.

Scientist Reviewer B

Strengths: The investigators' proposed use of 3RP-NF addresses the clinical needs of the population with NF including evidence that adults with NF have lowered QoL, as well as greater rates of anxiety and depression. Many studies have documented empirical support for 3RP in other disease groups (as referenced in the application), and the PI's own research has provided preliminary evidence of its efficacy for individuals with NF. The PI's other studies have logically proceeded in such a way that the intervention has been modified, tested, and validated for use with the population with NF, with the proposed large-scale clinical trial as the culmination of her previous work. As such, her current proposed intervention is consistent with psychosocial interventions considered standard of care in other disease groups. Regarding data collection measures, the majority of the measures proposed have appropriate psychometric properties (ie, adequate reliability and validity) and have been used with other populations with chronic illness. Furthermore, 2 of the measures have been endorsed for use in NF clinical trials by the patient-reported outcomes (PRO) group of the Response Evaluation in Neurofibromatosis and Schwannomatosis (REINS) committee.

Weaknesses: On a minor note, the Patient's Perception of Improvement Scale is a nonvalidated measure and consists of only one question regarding the patient's perceived improvement. As the question is only asked once and framed in a general way, this does not allow for the participant to differentiate ways the intervention helped (for example, if the participant saw improvement in depression but not pain, it is not clear how he/she would answer that question).

Research Strategy
Average Score: 9.0

Scientist Reviewer A

The PI will be studying an NF-specific targeted version of the 3RP intervention. Once participants enroll in the study, they will be randomly assigned either to the 3RP-NF intervention or HEP-NF. They will engage in an 8-week program with weekly meetings that focus on relaxation response strategies, cognitive behavioral training, positive psychology, and mind-body

interactions. The sessions will be carried out via video telemedicine. All outcomes will be measured through self-report via the secure Research Electronic Data Capturing (REDCap) system, which they have used in their NF studies in the past. There are 4 major assessment times including baseline, postintervention (8 weeks), 6, and 12 months. The outcomes target QoL, pain intensity, pain interference, depression, anxiety, and patient perception of improvement.

Strengths: This study demonstrates a high level of rigor in the design and methodology, which stems from the PI’s prior experience with this intervention and delivery method. The scientific rationale for testing this intervention is strong, based on a substantial amount of preliminary work the researchers have done in NF and more broadly with other patient populations. This is a team of researchers that are well established in this intervention methodology, as well as with this delivery method (telemedicine). There are clear study aims and objectives and clear hypotheses. The study methodology and data analytic approaches are clear and align with the objectives of the study and are expected to allow for successful study. This study includes all of the neurofibromatoses (NF1, NF2, schwannomatosis), which is certainly a strength. The focus of this intervention is not exclusive to one disease group over another; focusing on improving resilience in individuals with chronic medical conditions is a strength because it does not preclude anyone from benefitting, as the target for improvement is individualized. The inclusion and exclusion criteria are appropriate and justified. The team has dealt with concerns about suicidality in this mode of intervention appropriately and appears to have a good plan for managing any report of suicidal ideation during the course of the study in a participant. The scales chosen are appropriate, and when available, follow the recommendations of the REINS PRO committee. The application provides methodology in terms of managing missing data. Linear mixed models are proposed in order to compare changes in primary and secondary outcomes from baseline to each of the 3 follow-up evaluations.

Weaknesses: No weaknesses were noted.

Scientist Reviewer B

Strengths: The research strategy is clearly presented and well organized. The scientific rationale is provided, including a review of the use of 3RP in other populations as well as preliminary data in the population with NF obtained by the PI in previous studies. In review of these studies, there is sufficient evidence to more broadly test this intervention in the population with NF. Furthermore, the study includes the use of an active placebo group, and there is evidence from the PI’s previous work for use of this specific intervention as well. Previous research suggests that this study will be feasible to complete, as other studies have been successful in using the videoconferencing medium and retaining participants. In general, the study aims and hypotheses are appropriate. In Aim 1, the PI will determine the efficacy of 3RP-NF compared to an active placebo group, with outcome measures including assessment of QoL, anxiety, depression, and pain. In Aim 2, the PI will examine mediator and modifiers of improvement to explain outcomes. The study design and analyses will answer the research questions proposed. The PI has appropriately limited the number of outcome measures completed by participants and ensured the safety of participants who endorse suicidal ideation/intent by obtaining the contact information for a friend or family member. Inclusion and randomization are appropriate for a clinical trial. The PI provides appropriate justification for the exclusion criteria.

Weaknesses: No weaknesses were noted.

Recruitment, Accrual, and Feasibility
Average Score: 8.9

Scientist Reviewer A

Strengths: The PI appropriately addresses the availability of participants. In fact, the team has had an overwhelmingly positive response to their pilot study with this intervention and mode of delivery, such that they have a waiting list from that study that will be contacted for enrollment in this study once it is able to open. In addition, they are leveraging the CTF to advertise this study, which will reach a wide range of potential participants in the community with NF. The mode of delivery also allows for anyone in any location who can connect to the Internet access to the study. The team also showed excellent retention in the previous pilot study. The team describes in detail their prior experience with retention and strategies for retention in this study that appear to be appropriate. It does not appear that participation in this study will in any way interrupt the daily life of the individual participating. The feasibility of this study is strong given the team's prior experience and expertise in this intervention and mode of delivery. They have successfully completed multiple prior studies, including the pilot to the study.

Weaknesses: A minor weakness is that a detailed contingency plan for slow accrual is not provided, likely because of the notable positive response to the pilot study with regard to interest from participants and the current wait list for this study.

Scientist Reviewer B

Strengths: Based on her experience with her preliminary RCT, the PI was able to demonstrate her success in recruiting and retaining subjects. The PI has commitment from the CTF to assist with recruitment, which will allow her access to individuals with NF across the country. As she is well connected within the community with NF and had positive outcomes with these methods of recruitment in the past, it appears she will be able to obtain the relatively large sample size proposed. The PI developed the intervention to have as little impact as possible on day-to-day lives of participants, including having groups meet on evenings and weekends. Subject retention is always a concern with this type of intervention. However, the PI has provided well-thought-out strategies for retaining subjects. She implemented these in her preliminary RCT with much success, as she had very high retention rates. Given these factors, the clinical trial is feasible as presented.

Weaknesses: One of the inclusion criteria is a self-reported sixth-grade reading level, which is appropriate given the reading level of materials. However, the application does not indicate how this is to be reported. In reviewing the demographics form, the participant is asked whether they have a learning disability and their highest level of education completed, but there are no screening questions about reading.

Statistical Plan
Average Score: 8.4

Procurement Sensitive Document
Do not copy or distribute without CDMRP written permission.

Scientist Reviewer A

Strengths: The statistical plan is well written, including the power analysis, the description of evaluating potential confounders, and the analyses for the primary and secondary aims. Aim 1, comparing the 3RP-NF intervention to the HEP-NF intervention, will be evaluated with linear mixed models, which will allow for change comparisons between baseline and all 3 follow-up evaluations (8 weeks, 6, and 12 months). Aim 2 targets mediators and moderators to the relationship between the intervention and outcomes (QoL, pain, psychological status), and regression models and repeated measures ANOVA are to be employed. The statistical plan overall appears to be appropriate to being able to answer the questions being posed in this research study.

Weaknesses: No weaknesses were noted.

Scientist Reviewer B

Strengths: The statistical plan is appropriate for the proposed study. The power analysis has been completed to account for attrition at 6- and 12-month follow-up. The PI also includes a plan for analyses should attrition be higher than expected. The statistical analyses proposed for both Aims 1 and 2 are appropriate and will be adequate to identify whether 3RP-NF improves subjects' reports on outcome measures as well as identify mediating and moderating factors.

Weaknesses: No weaknesses were noted.

Biostatistician Reviewer

Strengths: Measurements and the times of measurements are clearly specified in the application. Statistical analysis for each endpoint is planned properly. The randomization procedure is clearly stated. Sample size and power calculations are carefully justified. Missing data are addressed. Attrition is discussed, and analysis is planned with attrition rate taken into consideration.

Weaknesses: Early-stopping rule is not planned for this trial. Although the research team proposes to monitor safety and study progress biannually, an independent data safety monitoring board (DSMB) is recommended to objectively review these tasks. It is unclear which time points of the possible mediators will be used in the mediation analysis. Although the rationales of the justification of measures are reasonable and clearly specified, the use of these justified measures has not been tested before, thus the validity of these measures is not confirmed.

Transition Plan
Average Score: 8.5

Scientist Reviewer A

Strengths: The study team presents multiple ways in which they will transition the information gleaned from this study to the broader scientific community and to the individuals affected by NF for care. Scientific dissemination will be done through presentations at the CTF annual conference and publications in peer-reviewed journals. There is a plan for training clinicians in this intervention with the support of CTF (marketing specialists) to allow for more access to

Procurement Sensitive Document
Do not copy or distribute without CDMRP written permission.

patients with NF to participate in the intervention in the future. The PI and Coinvestigator (co-I), Dr Park, have delivered training to clinicians in the past successfully. There is a plan to deliver this intervention as part of medical care at the PI's institution within a year of completing this study. They are also currently working on studies with adolescents with NF as well as adapted interventions for individuals with hearing impairment (NF2) that include live video and Computer Adaptive Real Time Translation (CART).

Weaknesses: The application indicates that clinical care via Vidyo (video telemedicine) is reimbursable by major insurance companies. With regard to the transition plan, this coverage is not consistent across states, and there are other factors when using this clinically that have to be considered (licensing of the practitioner in the jurisdiction that the patient resides in, etc). The lack of coverage by insurance companies could limit the extent that this intervention could be disseminated.

Scientist Reviewer B

Strengths: The PI states the goals of the proposed study as to (1) widely distribute access to the research and data and (2) make the intervention available to individuals with NF. She provides an organized plan to obtain these goals. The PI has an excellent publication record and will likely have no difficulty publishing in relevant, high-impact journals and presenting at meetings. In terms of lay presentations, the PI has already presented preliminary findings in formats geared toward the general public and, through these, has connections with media outreach both through the CTF and her local institution. Most impressively, the PI has a plan to train others in providing 3RP-NF. This is incredibly important, as finding that the intervention has benefit will be relatively meaningless if there is no way to provide it to individuals with NF. Partnering with CTF will allow the PI to reach NF providers across the country. The PI proposes several ways of training providers, including videoconferencing and in-person training, and she has provided herself protected time to apply for funding to provide such trainings. As such, the PI has an appropriate plan to transition from clinical trial findings to delivery of the intervention.

Weaknesses: No weaknesses were noted.

Personnel and Communication
Average Score: 9.9

Scientist Reviewer A

The PI, Ana-Maria Vranceanu, obtained her PhD in 2007 in clinical health psychology from Kent State University. She is currently an associate professor of psychology in the Department of Psychiatry at Harvard Medical School and assistant director for behavioral health integration in the Department of Psychiatry at MGH.

Strengths: The PI is Dr Vranceanu, a psychologist who has a strong academic, scientific, and clinical background. She has been the PI on 10 prior research grants. She and Dr Elyse Park, one of her co-Is, contributed to the development of the 3RP intervention and adapted it for patients with NF. She developed the HEP-NF as well. She provides this intervention clinically and has done so via live video. She has a strong relationship with the CTF, who has supported her prior work in this area. She has over 80 publications in peer-reviewed journals. The co-Is for this study

are Dr Park (psychologist/collaborator) and Scott Plotkin (MD, PhD). Dr Park is a senior R01-level researcher with 20 years of experience. She is the chief of behavioral research and an expert in mind-body research. She has over 150 publications. Dr Plotkin is a renowned expert in the NF field and a PI on multiple research grants. He is the director of the NF Clinic at Massachusetts General Hospital (MGH) and has over 100 publications. The study employs a biostatistician (Dr Eric Macklin), who has also been involved in multiple research projects and has worked with Dr Vranceanu on the pilot trials of this intervention. The study team has been operating together on this specific line of research for some time and appears to have a very productive, successful working relationship. The composition of the study team is very appropriate. This is a highly experienced team in NF generally, but more specifically in this intervention. The levels of effort appear appropriate. All logistics for this study are well circumscribed and meet the needs of the proposed clinical trial. They have demonstrated success with these specific logistics in the pilot study and other similar intervention trials.

Weaknesses: No weaknesses were noted.

Scientist Reviewer B

Strengths: The research team clearly has the experience and capabilities to successfully carry out the study. Indeed, the PI is likely the only person in the field of NF research qualified to do this type of clinical trial. The PI has assembled an excellent team with which to complete the study. The PI and Dr Plotkin are well known within the field of NF. Dr Park has many years of experience as a researcher and has worked on several trials of 3RP. The PI also has a biostatistician identified. While the PI has yet to identify the 2 clinicians, given her experience with conducting trainings in 3RP, there are no concerns with her ability to hire and train high-level clinicians. All team members have appropriate effort designated for the project, and appropriate plans are in place regarding communication and standardization of procedures.

Weaknesses: No weaknesses were noted.

UNSCORED EVALUATION CRITERIA

Ethical Considerations

Scientist Reviewer A

There are several factors associated with risk that have to be considered in this study, and the PI appears to have adequately addressed these risks, minimizing them for participants. The first is the security of the video telemedicine delivery approach to the intervention. The study will employ the use of a HIPPA-compliant system (Vidyo), which minimizes risk to breach of confidentiality of participants. There is a stated plan in the consent process to inform participants about maintaining confidentiality of fellow participants, which is standard procedure in this matter. Finally, the PI has adequately addressed a concern about potential suicidal ideation/plans in a participant and how to appropriately monitor and respond to such instances if they occur. The team’s prior research has shown these procedures to be consistent with sound research design, and these are being used in clinical practice by this team already as well. The informed consent process is also described and is appropriate to protect the participants.

Procurement Sensitive Document
Do not copy or distribute without CDMRP written permission.

Scientist Reviewer B

The PI has designed the study to have little risk for participants. The intervention itself has little risk, and there is a large body of evidence for its use. Appropriate safety plans are in place should suicidal ideation or intent be endorsed by a subject. The videoconferencing system chosen is Institutional Review Board (IRB)–approved and, as yet, has not had any security breaches. Privacy issues within the group therapy setting are addressed within the application and are appropriate, including concerns about confidentiality over social media; this is especially important given the relatively small community of individuals with NF. Consent procedures are appropriate.

Bioethicist Reviewer

The selection of subjects is equitable. Procedures are consistent with sound research design and, when appropriate, procedures are already in use for diagnostic or treatment purposes. Risks are minimized by use of key exclusion criteria. Risks are reasonable to any potential benefits to subjects and to society. There are adequate provisions to protect the privacy of subjects and to maintain the confidentiality of data. The informed consent form is exemplary. There are no vulnerable groups. However, there does not appear to be a research monitor (as required by DOD regulations) or a data-monitoring plan.

Environment

Scientist Reviewer A

This is a strong and supportive scientific environment. Besides MGH being a primary research institute, within that the Benson-Henry Institute for Mind-Body Medicine, a behavioral medicine service is well equipped to continue to support this study and study team. The collaboration between key study members is strong, and there is a strong connection with the community with NF in Dr Vranceanu and Dr Plotkin. MGH has a telepsychiatry virtual visit program that already supports clinical care through video teleconferencing with patients, and they have the technological support necessary for providing services in this manner. The biostatistics center is involved in this study and has the ability to fully support the analysis required in this study. Other facilities are appropriate for the support of this study. There is clear evidence of support from the institutions (MGH and CTF).

Scientist Reviewer B

The PI has successfully completed a pilot version of the current study at MGH, demonstrating that MGH is supportive of such research. A large academic institution such as MGH is clearly appropriate for the study, with all the necessary resources. The PI also has appropriate collaborations in place.

Procurement Sensitive Document
Do not copy or distribute without CDMRP written permission.

Budget

Scientist Reviewer A

The budget appears to be appropriate for the proposed clinical trial and is within the limits of the program announcement.

Scientist Reviewer B

The budget is appropriate and includes all necessary personnel and materials.

Application Presentation

Scientist Reviewer A

This is an extremely well-written, well-developed protocol. The writing, clarity, and presentation of the application is positive and had a positive impact on the evaluation as it made it clear this is a well-thought-out application, and much care was taken to develop the application and address all areas appropriately.

Scientist Reviewer B

The application is organized, clear, and well written. While there are a few “spell-check typos,” this does not interfere with the clarity of the application.

Community Needs

Scientist Reviewer A

The PI and co-I, Dr Plotkin, are well-respected clinicians and researchers in the community with NF. They are also highly active in the clinical care of individuals with NF. They are known to have a high level of understanding and care of the needs of individuals with NF, and this application reflects this understanding and knowledge.

Scientist Reviewer B

The PI has been active within the community with NF and clearly knows its needs. She shows great respect for the community by proposing a project that individuals with NF deem important and unrecognized by research. She approaches the project with safeguards in place for individuals who may have learning issues or other problems related to NF. She also recognizes that this project will omit some portion of the community with NF (for example, those with deafness) but has plans to include them in future research, once the intervention is validated further. The PI approaches this study in a knowledgeable and respectful manner.

Consumer Reviewer

To enhance QoL through a mind-body treatment is a unique, original, and attainable goal for patients with NF. Patients will be informed that there may be no benefit from this trial, and some may become better able to cope with the symptoms and treatments of NF while increasing their

Procurement Sensitive Document
Do not copy or distribute without CDMRP written permission.

self-awareness and self-esteem. This study therefore reflects the needs of the patient with NF and respects their knowledge.

Procurement Sensitive Document
Do not copy or distribute without CDMRP written permission.
